# Awareness of polycystic ovary syndrome: A university students’ perspective

**DOI:** 10.1016/j.amsu.2021.103123

**Published:** 2021-12-04

**Authors:** Eman Alshdaifat, Amer Sindiani, Zouhair Amarin, Nadine Absy, Noor AlOsta, Husam Aldean Abuhayyeh, Mustafa Alwani

**Affiliations:** a Department of Obstetrics and Gynecology, Faculty of Medicine, Yarmouk University, Irbid, Jordan; b Department of Obstetrics and Gynecology, Faculty of Medicine, Jordan University of Science and Technology, Irbid, Jordan; c Faculty of Medicine, Jordan University of Science and Technology, Irbid, Jordan; d Prince Hamza Hospital, Amman, Jordan

**Keywords:** Attitude, Awareness, Knowledge, Polycystic ovary syndrome, Women's health

## Abstract

**Background:**

Polycystic ovary syndrome is the most common endocrine disorder in women of reproductive age. Women with this syndrome may have infrequent menstrual periods or amenorrhea and excess androgen levels. The ovaries develop numerous small follicles and fail to ovulate on a regular basis, with subsequent subfertility in those women that wish to conceive.

The etiology of polycystic ovary syndrome is unclear. Early diagnosis and treatment may reduce the risk of long-term complications such as type 2 diabetes and heart disease.

**Objectives:**

To assess the knowledge and attitude of university students towards polycystic ovary syndrome at two universities in the north of Jordan.

**Method:**

ology: This is a cross-sectional online survey that polled female students at two universities in the north of Jordan. The main outcome measures included average polycystic ovary syndrome awareness score, predictors of high awareness scores, and sources of information.

**Results:**

Formal diagnosis of polycystic ovary syndrome was reported by 29.9% of the 1182 students, the average polycystic ovary syndrome awareness score was M = 11.59 (SD = 4.95). Being a 6th and 5th year college student were the strongest independent predictors for recognizing the term polycystic ovary syndrome, in addition to be a student in the majors of veterinary medicine, nursing, pharmacy, or dentistry. Age was a significant predictor of polycystic ovary syndrome awareness score. Being investigated for, or being diagnosed with polycystic ovary syndrome were significant predictors of higher scores. Body mass index was a weak predictor of polycystic ovary syndrome awareness. Participants who reported to have hirsutism, acanthosis nigricans, or acne scored significantly higher than others.

Healthcare professionals were the most common source of information reported by participants. Lectures were most effective in increasing awareness score but were poorly utilized outside the curriculum.

**Conclusion:**

Although students in this study demonstrated a satisfactory level of polycystic ovary syndrome awareness and were more likely to seek information from healthcare professionals, this level of awareness should spread-out to other segments of the population.

## Highlights

• Medical field students were highly cognizant about PCOS.
• Higher educational years has higher awareness regardless of age.
• No significant difference between diagnosed and free PCOS groups in awareness score.

## 1 Introduction

Polycystic ovary syndrome (PCOS) is a heterogeneous disorder that leads to the overproduction of androgens mainly from the ovaries. The syndrome is associated with insulin resistance and is diagnosed by the presence of certain clinical, biochemical, and ultrasonographic criteria [1].

The etiology of PCOS remains unknown, although it is hypothesized that certain genetic factors contribute to its pathophysiology, where those with a genetic predisposition are more likely to express features of PCOS when exposed to certain environmental conditions [2].

A wide range of clinical presentations are attributed to PCOS which include, but are not limited to, symptoms associated with hyperandrogenemia such as hirsutism, acne, abnormal unintended weight gain, male pattern alopecia, symptoms related to anovulation including irregular heavy menstruation, oligomenorrhea, amenorrhea and subfertility, in addition to symptoms resulting from insulin resistance such as darkening of skin folds and dyslipidemia [3].

Diagnosis is usually made when a patient has two of three main features, or their associated phenotypes, that include hyperandrogenism, polycystic ovaries and anovulation [4].

Early detection and therapy of this disorder would decrease its associated long-term effects. About 40–80% of patients with PCOS are either overweight or obese, thus increasing the risk of metabolic syndrome, endometrial hyperplasia and endometrial cancer [[5], [6], [7]].

Polycystic ovary syndrome remains an underdiagnosed condition [8] despite the fact that it represents the most common endocrine syndrome in women of reproductive age [2].

Studies have found that there was a gap in the knowledge of students about PCOS and its symptoms and signs, and that lifestyle preferences may predispose to PCOS [9]. Delay in the diagnosis of PCOS may lead to metabolic and reproductive abnormalities associated with it [7]. other studies have found that the prevalence of having signs and symptoms of PCOS was on the increase without a change in the level of awareness among female students, even though many of them had suffered from the syndrome. In addition, most of the students do not visit their doctors when suffering from PCOS symptoms and signs [10].

This study aimed at assessing the knowledge and attitude of university students towards PCOS at two universities in the north of Jordan.

## 2 Materials and methods

### 2.1 Design and setting

This is a cross-sectional study. Data were collected between 22nd February and 6th

March 2021 through an online questionnaire using Google Forms. The questionnaire was constructed by four gynecologists and one statistician. Questions and answers were written in both Arabic and English. The questionnaire covered participants' age, marital status, education level, monthly income, smoking status, and body mass index (BMI) (File 1). A pilot study on 30 randomly selected students was conducted and their responses were omitted from the final analysis. The questionnaire was uploaded on Google forms and distributed to medical students, through social media platforms and students’ university emails.

Inclusion criteria were being a current female student, residing in Jordan and aged 18 and above. A short paragraph about the study was shared using a Uniform Resource Locator (URL) link to the questionnaire, and contained a consent form for participation in the study and the emails of the researchers to answer any inquiries. Reminders to fill out the questionnaire were sent on day seven and day ten.

Participants’ awareness about PCOS was measured by their ability to identify PCOS causes (2 points), symptoms (8 points), diagnostic workup (2 points), treatment (7 points), and associated conditions (3 points). For example: “*Identify the causes of PCOS (tick all correct answers):*

Genetics
Hormonal
Others
*I do not know*”

Participants who answered “genetics” and “hormonal” were correct. Those who chose both scored 2 points, and one point for choosing one of them, and zero for not choosing any of them. Participants who chose “I do not know” and another answer were omitted for giving contradictory responses.

A total score of 22 points was calculated and was assumed to represent the overall awareness of participants about the disease.

Regarding familiarity with PCOS, participants were asked whether they could recognize the term PCOS, source of information, and if they knew someone with the condition. For self-reported PCOS status and symptoms, participants were asked about cycle frequency, hirsutism, acne, acanthosis nigricans, and if they have ever been investigated for PCOS by ultrasound, gonadotropins levels and androgens.

### 2.2 Sample

Of the 1278 responses to the questionnaire, 22 were omitted due to duplication, and 74 due to contradictory answers. The remaining 1182 respondents were further grouped according to age, marital Status, university, year of study, and monthly income (Table .1).

**Table 1:** Participants' Data

	count	Average score (95% CI)	ANOVA or *t*-test *p*
Age			
18-19	377	9.9 (9.5–10.4)	<0.001
20-21	427	11.7 (11.3–12.2)	
22-23	238	13.7 (13.1–14.3)	
24 and above	140	12.0 (11.2–12.8)	
Marital status			
Single	1098	11.0 (7.9–14.1)	0.612
Married	73	12.1 (11.0–13.3)	
Divorced	11	11.6 (11.3–11.9)	
University			
JUST	715	12.3 (11.9–12.6)	<0.001
Yarmouk	467	10.6 (10.1–11.0)	
Residency			
North of Jordan	883	11.4 (11.1–11.8)	0.201
Middle of Jordan	258	12.0 (11.4–12.6)	
South of Jordan	41	12.3 (10.8–13.9)	
Income			
<500	387	9.9 (9.4–10.4)	<0.001
500-1000	474	12.0 (11.6–12.4)	
>1000	321	13.1 (12.5–13.6)	
Year of studying			
First year	187	8.8 (8.1–9.5)	<0.001
Second year	232	10.6 (10.0–11.2)	
Third year	208	11.2 (10.6–11.8)	
Fourth year	198	11.8 (11.1–12.4)	
Fifth year	114	14.8 (14.0–15.6)	
Sixth year	75	17.0 (16.3–17.8)	
Higher education	168	11.7 (11.0–12.4)	

### 2.3 Statistical analysis

For Statistical analysis, the responses were transferred to an Excel spreadsheet. The SPSS version 20 software was used. T-tests, ANOVA, ANCOVA, and regression analysis were utilized. Variables were converted to dummy form when appropriate. Single variables were used to measure their association with PCOS scores, followed by analysis of confounders. Independent and statistically significant predictors of the total awareness score were then analyzed in a final multivariate linear regression model by using the Enter method (Table 5). Alpha level of <0.05 was considered as significant.

### 2.4 Ethical consideration

This study was approved by the Deanship of Research and the Institutional Review Board (IRB number: 35/137/2021) of the Jordan University of Science and Technology and was registered at ResearchRegistery.com (Unique Identifying Number: researchregistry7247) [17] in accordance with the declaration of Helsinki. This article has been reported using STROCSS criteria [18].

## 3 Results

Out of 1182 respondents, 377 (31.9%) were 18–19 years of age, 427 (36.1%) were 20–21, 238 (20.1%) were 22–23, and 140 (11.8%) were 24 or older. A formal diagnosis of PCOS was reported by 353 (29.9%) participants. Most participants were students at the Jordan University of Science and Technology (n = 715, 60.5%).

Regarding Participants ‘financial status, 387 (32.7%) reported a monthly income of less than 500 Jordanian Dinars (JD), 474 (40.1%) between 500 and 1000, and 321 (27.2%) participants reported more than 1000 JDs (1 JD = 1.41 $). The demographics and students' academic level distribution is shown in Table 1.

The average of PCOS awareness score was M = 11.59 (SD = 4.95). The minimum score was 0/22 (n = 21) and maximum score was 22/22 (n = 6).

Age groups differed significantly in their PCOS awareness scores (p < 0.001), and the highest scoring group was in those aged between 22 and 23 years old (Table 1). The variability between age groups in terms of scores was no longer significant after accounting for academic level as a covariate in the analysis (p = 0.989).

**Fig. 1:**
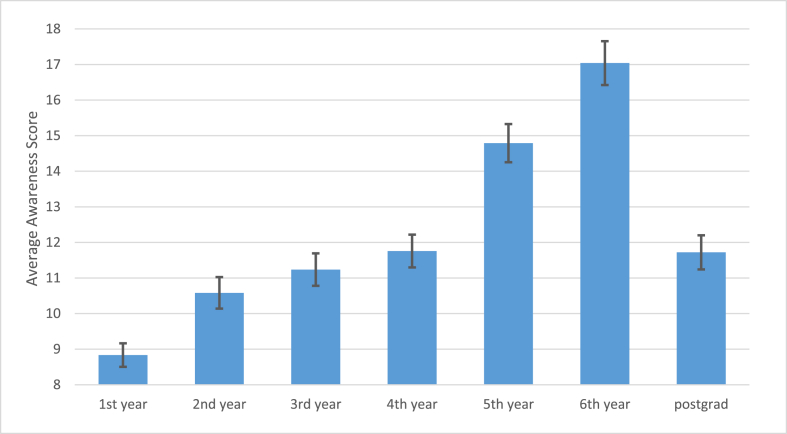
Average scores as predicted by years in university. Error bars represent the standard error.

The participants’ scores at different academic levels were significantly different (p < 0.001) (Fig. 1). In general, a higher academic level was associated with a higher score.

Additionally, Participants who were diagnosed with PCOS (n = 353, M = 13.55, SD = 4.04) scored significantly higher than those who were not (n = 829, M = 10.76, SD = 5.07, p < 0.001).

Participants who reported being investigated for PCOS by abdominal US, gonadotropins, or androgens scored significantly higher than those who reported not having been tested or who were not sure if they were tested (Table 2).

**Table 2:** Participants' being investigated.

	Abdominal Ultrasound			Gonadotropins			Androgens		
	Yes	No	I am not sure	Yes	No	I am not sure	Yes	No	I am not sure
Count	500	655	27	429	669	84	323	797	62
Mean	13.50	10.21	9.81	13.83	10.37	9.90	14.38	10.48	11.42
SD	4.22	5.01	4.56	3.95	5.07	4.61	3.59	5.04	4.25
ANOVA	F (2,1179) = 72.201, p < 0.001			F (2,1179) = 77.824, p < 0.001			F (2,1179) = 80.972, p < 0.001		
Post hoc *p*	<0.001	Ref	0.903	<0.001	Ref	0.660	<0.001	Ref	0.276

Participants’ responses on having PCOS symptoms are summarized in Table 3. Having hirsutism, acanthosis nigricans or abnormal menstrual cycle frequency were significant or marginally significant predictors of higher scores after accounting for the investigated variables as confounders.

**Table 3:** PCOS symptoms.

	Irregular Cycle				Hirsutism				Cycle Frequency		
	Never	Few times	Many times	Always	Never	Few times	Many times	Always	<22 days	22–40	>40 days
Count	275	409	284	214	399	288	218	277	156	902	124
Mean	10.92	11.25	11.77	12.90	10.98	10.92	11.69	13.10	9.74	11.72	13.05
SD	5.21	4.88	4.81	4.73	5.12	4.99	4.58	4.63	4.93	4.94	4.40
ANOVA	F (3,1178) = 7.550, *p* < 0.001				F (3,1178) = 12.723, *p* < 0.001				F (2,1179) = 17.024, *p* < 0.001		
Post hoc *p*	Ref	0.821	0.171	<0.001	Ref	0.999	0.316	<0.001	<0.001	Ref	0.013
ANCOVA	F (3,1172) = 0.415, *p* = 0.742				F (3,1172) = 2.852, *p* = 0.036				F (2,1173) = 9.871, *p* < 0.001		

	Acne				Acanthosis Nigricans			
	Never	Few times	Many times	Always	Never	Few times	Many times	Always
Count	206	362	324	290	700	273	108	101
Mean	11.02	11.65	11.78	11.72	11.54	11.08	11.79	13.15
SD	5.11	4.98	4.88	4.89	4.94	5.11	4.52	4.86
ANOVA	F (3,1178) = 1.158, *p* = 0.325				F (3,1178) = 4.411, *p* = 0.004			
Post hoc *p*	Ref	0.456	0.315	0.401	Ref	0.557	0.963	0.012

Students’ scores in different college majors are presented in Table 4. Linear regression analysis was undertaken for scores in different college majors as a predictor of PCOS awareness score, Islamic studies acted as a reference group. The least significant group was removed from the model until the remaining groups in the model (n = 9) were significantly higher than the reference group. The model had R2 = 0.161, F (9, 1172) = 25.077, p < 0.001. These remaining 9 groups were used as predictors in the final linear regression model.

**Table 4:** Participants' speciality.

	Count	Mean		Count	Mean
Medicine	240	14.48	Mass communication	18	10.11
Pharmacy	103	13.54	Law	27	9.81
Agriculture	34	13.21	Economics and administrative science	47	9.64
Dentistry	51	12.78	Fine Art	12	9.58
Veterinary medicine	10	12.70	Information technology and computer science	69	9.39
Nursing	62	11.90	Archaeology and anthropology	11	9.36
Medical applied sciences	108	11.56	Education	105	8.93
Sciences	118	10.80	Tourism and Hotel management	5	8.00
Engineering	135	10.29	Shari'a and Islamic studies	27	7.96

**Table 5:** Final Linear regression model for all study parameters.

Predictor	Coefficient (95% CI)	P value
Constant	2.32 (0.96–3.68)	0.001
Investigated by US		
No	Ref	
Yes	0.67 (0.11–1.22)	0.018
Not sure	- 0.44 (- 1.93–1.05)	0.563
Investigated by gonadotropins		
No	Ref	
Yes	0.69 (- 0.06–1.44)	0.071
Not sure	- 0.14 (- 1.09–0.81)	0.768
Investigated by androgens		
No	Ref	
Yes	1.38 (0.58–2.17)	0.001
Not sure	0.32 (- 0.78–1.42)	0.569
Study year		
First year	Ref	
Second year	0.88 (0.15–1.62)	0.019
Third year	0.97 (0.21–1.72)	0.012
Fourth year	1.61 (0.83–2.39)	<0.001
Fifth year	2.82 (1.84–3.81)	<0.001
Sixth year	4.45 (3.25–5.64)	<0.001
Postgraduate	1.52 (0.69–2.36)	<0.001
Income		
< 500 JDs	Ref	
500–1000 JDs	0.97 (0.45–1.50)	<0.001
>1000 JDs	0.32 (- 0.31–0.96)	0.318
Familiarity		
Know someone diagnosed; No	Ref	
Know someone diagnosed; Yes	0.82 (0.26–1.38)	0.004
Know someone diagnosed; Not sure	- 0.56 (- 1.33–0.21)	0.155
Recognize the term PCOS	3.65 (2.48–4.82)	<0.001
Sources of knowledge		
Doctors	1.10 (0.60–1.59)	<0.001
Lectures	1.82 (1.13–2.50)	<0.001
Others	0.82 (0.26–1.38)	0.004
Have Hirsutism		
Never	Ref	
Few times	0.05 (−0.53–0.63)	0.863
Many times	0.13 (- 0.53–0.79)	0.697
Always	1.36 (0.73–2.00)	<0.001
Menstrual cycle frequency		
< 22 days	- 0.82 (- 1.47 – - 0.17)	0.014
22–40 days	Ref	
> 40 days	- 0.04 (- 0.79–0.70)	0.906
College major		
Veterinary medicine	2.48 (0.08–4.87)	0.042
Nursing	2.46 (1.38–3.54)	<0.001
Medicine	2.28 (1.45–3.11)	<0.001
Pharmacy	2.15 (1.25–3.05)	<0.001
Dentistry	1.76 (0.58–2.93)	0.003
Medical applied sciences	1.49 (0.64–2.34)	0.001
Agriculture	1.48 (0.11–2.85)	0.035
Natural sciences	1.27 (0.47–2.07)	0.002
Engineering	0.48 (- 0.29–1.26)	0.220
All other majors	Ref	

Of all participants, 1133 (95.9%) recognized the term PCOS and scored significantly higher than those who did not (p < 0.001, M = 11.85, SD = 4.79 vs M = 5.61, SD = 4.91). Participants who reported to know someone diagnosed with PCOS (n = 786, 66.5%, M = 12.32, SD = 4.65) scored significantly higher than those who reported otherwise (n = 239, M = 10.64, SD = 5.35; P < 0.001), or who were not sure (n = 157, 13.3%, M = 9.42, SD = 4.94; p < 0.001). These two variables remained significant predictors after analysis for confounders.

Higher income was associated with significantly higher scores and was not subjected to confounders. Marital status and area of residence were not significant predictors of PCOS awareness score (Table 1).

The mean BMI was 23.88 (SD = 4.68). Body mass index was a significant but weak predictor of score (F (1, 1180) = 36.717, R^2^ = 0.030, p < 0.001). Smoking status was not associated with significant difference in awareness score (p = 0.437).

Regarding the source of knowledge, 465 participants reported doctors as their source of knowledge, 285 university courses and lectures, 283 friends, 425 media, 378 relatives, and 261 reported other sources. When running multiple regression analysis for these variables, the model predicted R^2^ = 23.8% of the variance in PCOS awareness score (F (6, 1175) = 61.092, p < 0.001). Further analysis shows only doctors (β = 2.78, p < 0,001), lectures (β = 4.30, p < 0.001), and other sources (β = 0.86, p = 0.006) were significant predictors of higher scores. When accounting for university majors and year of study, lectures coefficient decreased to (β = 1.53, p < 0.001) whereas doctors and other sources were minimally affected. When accounting for being investigated for PCOS in the analysis, doctors as a source of knowledge coefficient decreased to (β = 1.44, p < 0.001), others decreased to (β = 0.30, p = 0.319), while lectures was minimally affected.

## 4 Discussion

Despite the unwavering evidence of PCOS deleterious effects, delayed diagnosis due to lack of awareness remains an issue [9,11]. A Saudi study showed that two thirds of 350 female participants (66.3%) had inaccurate knowledge about the risks of PCOS due to a lack of discussions regarding reproductive health in schools and families [12]. This may be attributed to the fact that reproductive health topics are not usually included in school curricula and the absence of easy access to resources. In this study, 98.9% of respondents felt that spreading awareness about the syndrome was important.

Knowledge acquired throughout university years was a better predictor than years of life, and was associated with higher awareness levels. In this study, those who were in their 5th and 6th year had the highest awareness scores, this could be attributed to the fact that medical and doctor of pharmacy students have six-year study programs, which is not the case in other majors, added to the fact that those two majors include PCOS in their curricula. An additional contributing factor may be that the more years spent at university the more students will be exposed to awareness campaigns and off-campus lectures. This finding is in line with a recent study about PCOS awareness in Jordan [13]. Another study conducted in Saudi Arabia concluded that the level of knowledge of PCOS was significantly proportional to higher educational level [14].

Regarding economic status, those who have higher monthly income scored significantly higher in comparison to those with a lower income. A study conducted in Iran to investigate the relationship between socioeconomic status and oral cancer awareness in adults had the same results; socioeconomic inequalities were observed and it was established that the higher the wealth index, the higher the awareness score [15].

Knowing someone who is diagnosed with PCOS was associated with higher awareness scores. In addition, Students studying medically-related majors at both universities, such as veterinary medicine, nursing, medicine, pharmacy, or dentistry, scored higher than those who study other majors.

Approximately 90%–95% of anovulatory women presenting to infertility clinics have PCOS [16]. The most commonly reported symptom among our participants was acne, which may be more of a cosmetic observation than a clinical symptom and is relatively common in adolescents and young adults. This may explain why it was not a good predictor of PCOS awareness. In addition, high awareness scores were observed among those who reported having hirsutism, whereas the least common symptom to be reported was acanthosis nigricans, but those who reported having it had the highest awareness scores among all other symptoms. It seems that the cosmetic signs of PCOS are more likely to prompt patients to probe into knowing more and to seek advice.

Regarding the sources of knowledge regarding PCOS in this study, similar findings were documented in other studies in which doctors were found to be the main source of information about PCOS [13]. When accounting for being investigated for PCOS, doctors as a source of knowledge coefficient decreased while doctors’ lectures were less affected.

The limitation of this study is being a cross sectional study with a convenience sampling technique using a newly developed questionnaire and scoring system. Score validation and nationwide longitudinal studies are required.

## 5 Conclusion

Students in this study demonstrated a good level of PCOS awareness and mainly sought information from healthcare professionals. It would be highly desired to spread awareness to other sections of the population at large.

## Ethical approval

This study was approved the Deanship of Research and the Institutional Review Board (IRB number: 35/137/2021) of the Jordan University of Science and Technology, and were registered at ResearchRegistery.com (Unique Identifying Number: researchregistry7247) [17] in accordance with the declaration of Helsinki.

## Sources of funding

No funding was granted.

## Author contribution

Study concept and design: Eman Alshdaifat, Amer Sindiani, Zouhair Amarin Data collection: Eman Alshdaifat, Nadine Absy, Noor AlOsta Data analysis: Husam Abuhayyeh Writing the paper: Eman Alshdaifat, Amer Sindiani, Zouhair Amarin, Nadine Absy, Noor AlOsta Final revision: Eman Alshdaifat, Amer Sindiani, Zouhair Amarin, Mustafa Alwani.

## Consent

A short paragraph about the study was shared using a Uniform Resource Locator (URL) link to the questionnaire and contained a consent form to participate in the study and the emails of the researchers to answer any inquiries.

## Registration of research studies

1. Name of the registry: Awareness of polycystic ovary syndrome: A university students' perspective.
2. Unique Identifying number or registration ID: researchregistry7247
3. Hyperlink to your specific registration (must be publicly accessible and will be checked): https://www.researchregistry.com/register-now#home/registrationdetails/61647ab06342f0001ee5456c/

## Guarantor

Eman Alshdaifat.

## Provenance and peer review

Not commissioned, externally peer-reviewed.

## Declaration of competing interest

Authors has no conflict of interest to be declared.
